# Bacterial Nanocellulose toward Green Cosmetics: Recent Progresses and Challenges

**DOI:** 10.3390/ijms22062836

**Published:** 2021-03-11

**Authors:** Tânia Almeida, Armando J. D. Silvestre, Carla Vilela, Carmen S. R. Freire

**Affiliations:** CICECO—Aveiro Institute of Materials, Department of Chemistry, University of Aveiro, 3810-193 Aveiro, Portugal; taniaralmeida@ua.pt (T.A.); armsil@ua.pt (A.J.D.S.); cvilela@ua.pt (C.V.)

**Keywords:** bacterial nanocellulose, green cosmetics, facial mask, skin active substances, carrier, cosmetic formulations

## Abstract

In the skin care field, bacterial nanocellulose (BNC), a versatile polysaccharide produced by non-pathogenic acetic acid bacteria, has received increased attention as a promising candidate to replace synthetic polymers (e.g., nylon, polyethylene, polyacrylamides) commonly used in cosmetics. The applicability of BNC in cosmetics has been mainly investigated as a carrier of active ingredients or as a structuring agent of cosmetic formulations. However, with the sustainability issues that are underway in the highly innovative cosmetic industry and with the growth prospects for the market of bio-based products, a much more prominent role is envisioned for BNC in this field. Thus, this review provides a comprehensive overview of the most recent (last 5 years) and relevant developments and challenges in the research of BNC applied to cosmetic, aiming at inspiring future research to go beyond in the applicability of this exceptional biotechnological material in such a promising area.

## 1. Introduction

Consumers are increasingly aware of the current issues about environmental pollution and sustainability that the world is facing. The depletion of natural resources, waste generation, climate changes, and water and air pollution are among the main challenges to be tackled [1]. There is also an increasing concern with healthcare and lifestyle that has changed consumer trends and is driving the demand for natural products which at the same time are expected to be more effective and safer, especially in sectors such as cosmetics and food [2,3]. The ambitious goals and targets for the next years set by the world authorities for environment and sustainable development, in the scope of the 2030 Agenda for Sustainable Development, are prompting countries to adopt more stringent environmental policies [4]. For instance, this involves regulating the production and sales of plastic bags and single-use plastics items, the phase-out of the intentional use of microplastics, the labeling of harmful ingredients or even the taxes applied to the waste management, including recycling [5,6]. These policies are essential to stimulate green development [7] that has also been influencing how companies are evolving and becoming “greener”. In particular, the cosmetic industry is an ever-growing sector that is among the most innovative and science-driven industries, in which we have also been witnessing a great effort to follow this global trend for natural, environmentally friendly and sustainable production and products [8]. The environmental impact of cosmetic products lies mainly in the extensive use of non-renewable and non-biodegradable raw materials [9]. An example of this is the broad use of plastic microbeads as decorative or exfoliator agents or the use of parabens as preservatives, silicones as emollients or emulsifiers, or even the inclusion of chemical and mineral UV filters in many cosmetic products [8]. Thus, since most cosmetics are used daily, the addition of these ingredients to the cosmetic formulation raises significant pollution concerns. In addition, the manufacturing process and the packaging are two other important stages/steps to consider in the environmental life cycle assessment (LCA) of a cosmetic product [8,10].

In this sense, there has been a growing investment by the cosmetic industry in developing green-tech solutions, either to replace synthetic ingredients by bio-based counterparts or to modify the production technology or even to redesign and develop recyclable and biodegradable cosmetic packaging materials [8,11].

In the “green” advent of the cosmetic industry, some biopolymers have gained high popularity not only because of their biodegradable and environmentally friendly nature but also due to their biocompatibility that categorize them as skin-friendly materials [12]. Biopolymers, such as proteins (e.g., collagen and wheat proteins) and polysaccharides (e.g., cellulose, alginic acid, and hyaluronic acid), are already widely used as ingredients in cosmetic formulations to ameliorate properties of the products (e.g., texture and consistency) or boost their action on the body (e.g., hydration and wrinkles reduction) [13]. Furthermore, currently, biopolymers are also recognized as a viable and sustainable alternative to petroleum-based polymers commonly used in cosmetic packaging [11].

Cellulose is the most abundant biopolymer worldwide, which together with its remarkable properties (e.g., hydrophilicity, extensive modification capability, and thermal stability up to about 200 °C) make it an attractive source of new sustainable materials for many industrial applications [14]. Cellulose is obtained mainly from plants, but it is also produced by bacteria, fungi, algae, and marine animals [15]. Irrespective of its source, it is a linear polysaccharide of β-d-glucopyranose units linked by β-1,4-glycosidic bonds [16]. Although plant cellulose is the most used cellulose substrate, bacterial nanocellulose (BNC, also known as microbial cellulose, bacterial cellulose or biocellulose), is currently regarded as a promising cellulose form for a wide range of applications, including in cosmetic [17,18], food [18,19,20], pharmaceutical and biomedical [21,22,23,24,25,26], and technological fields [27,28,29,30].

BNC is a nano scale form of cellulose that is biotechnologically produced as an exopolysaccharide by some aerobic non-pathogenic bacteria (e.g., *Komagataeibacter* (formerly *Acetobacter*), *Agrobacterium*, *Aerobacter*, *Achromobacter*, *Azotobacter*, *Rhizobium*, *Sarcina*, *Salmonella,* and *Escherichia*), which combines the properties of cellulose with the features of nanomaterials [31,32].

The applicability of BNC in cosmetics has been explored and demonstrated over the years, especially as a support material of sheet facial masks for the skin delivery of active substances. However, BNC has also been employed in natural scrub cosmetics or as a structuring agent in personal care formulations [18,21]. In fact, some BNC-based cosmetic products are already available in the market, which are many times advertised as biocellulose and associated with ingredients that frequently place these products in the group of “luxury” cosmetics [33]. However, its application is still limited despite its recognized high-performance and unique set of properties. However, considering the growth forecasts for the personal care market, which is expected to grow at a compound annual growth rate (CAGR) of about 5.0% over the next five years [34], and the high demand for bio-based products, it is foreseen that the research and commercial interest on the application of BNC in green cosmetics will greatly increase in the near future. This was also envisaged in a very recent bibliometric appraisal covering the applicability of BNC in cosmetics in which it is shown that the number of publications and interest on this topic have been gradually increasing in recent years [17]. This study also emphasizes the reduced number of detailed bibliographic reviews on this subject that might provide an overview of the actual status and the research trends that will foster new ideas [17]. Hence, in the current review, the most recent and relevant investigation results and developments on BNC applied to cosmetics are comprehensively systematized. From this perspective, not only research articles but also patents will be covered, because although registered patents not always result in commercial products, they are a good indicator of market trends and of further directions for research and investment [35]. The challenges that are hampering a faster progress of the commercial application of BNC and future perspectives are also highlighted, aiming to spur future research and go further in the application of this exceptional biopolymer in such a promising area.

## 2. Biosynthesis and Production Methods of Bacterial Nanocellulose

Among BNC-producing bacteria, those from the *Komagataeibacter* genus are recognized as the most effective [36]. *Komagataeibacter* bacteria belong to the acetic acid bacteria group and are commonly present in sugar-rich beverages and fruit products and residues [36]. In particular, the species *Komagataeibacter xylinus,* owing to its capability in using a wide range of sugars and the higher production rates of BNC, is considered a microbial model to study the BNC biosynthetic pathway [37]; more recently, the *Komagataeibacter hanseiin* is also capturing increasing interest as a model microorganism, especially because it is also a high-yield BNC producer [16,38]. In addition to these species, others have also demonstrated high productivities, being also recognized as efficient BNC producers, namely, the *Gluconacetobacter sacchari* isolated from Kombucha tea [39] or the *K. rhaeticus* (strain PG2) isolated from pomegranate [40].

The biosynthesis of BNC is a well-concerted and highly regulated process involving multiple enzymes and complexes of catalytic and regulatory proteins that can be divided in two main stages: first, the production of β-1,4-glucan chains, and then the assembly and crystallization of cellulose [41]. Succinctly, the process initiates with the transport of the carbon source (e.g., glucose and fructose) into the cell, where the cellulose precursor uridine diphosphoglucose (UDPG) is formed. Then, glucose is polymerized from UDPG into β-1,4-glucan chains by cellulose synthase. Finally, the cellulose chains are secreted through pores in the cell membrane as sub-fibrils, which are further assembled into ribbons organized in a 3D nanofiber network stabilized by hydrogen bonds [41,42]. The biosynthetic pathway of BNC and associated mechanisms were addressed in detail in previous reviews [37,41]. More recently, Jacek et al. [16] reviewed the latest advances regarding the molecular aspects behind BNC biosynthesis.

**Figure 1:**
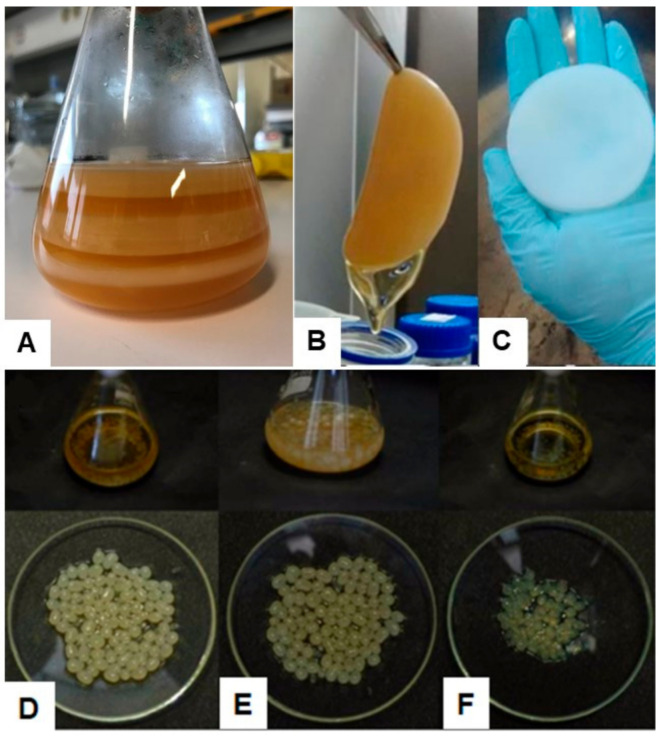
Bacterial nanocellulose shapes: bacterial nanocellulose (BNC) membranes produced in static fed-batch conditions (**A**); wet BNC membrane produced in static culture in Hestrin–Schramm (HS) medium, before purification (**B**) and after purification (**C**) (adapted with permission from [52]); BNC spheres produced under agitated conditions using mannitol (**D**), glucose (**E**), and xylitol (**F**) as carbon source (reprinted with permission from [53]).

BNC is the result of an oxidative fermentation process, with the oxidation of sugars and organic acids that can be produced either in synthetic or non-synthetic sugar and nitrogen-rich media at temperatures ranging between 25–30 °C and pH 4.5–7.5 [43]. There are two general methods for BNC production: static culture (in flasks or trays) and agitated culture (in jar fermenters). The most used method at a laboratory scale is the static culture, in which BNC is accumulated as a gelatinous membrane (hydrogel-like) (Figure 1A–C) at the air–medium interface, which thickens with culture time [14,44]. The formation of the floating BNC membrane helps bacteria access oxygen in the surface medium, and it is generally accepted that it functions as a barrier to protect bacteria from radiation, natural inhibitors, and drying [41,42]. A static fed-batch methodology may also be used by the periodic addition of culture medium with the formation of a new BNC membrane each time, thereby increasing the expected production [45,46]. The traditional static culture still offers the possibility of placing a mold in the culture medium to produce a BNC membrane with defined shapes, which is favorable for applications with a required predefined form [20]. Despite being widely used at the laboratory scale and by some traditional industries (producers of nata de coco, mainly in Asia), static culture has some limitations that still hinder its broader use at the industrial level. Specifically, it is labor-intensive, needs high amounts of culture medium, has a long cultivation time (several days), and has low productivity, which make it a high-cost and inefficient method [47,48].

Alternatively, in agitated culture, BNC is generated in variable shapes and sizes, namely fibrous suspension, irregular masses, pellets, or spheres (Figure 1D–F), depending on various factors (e.g., rotating speed, carbon source, and culture time) [44,49]. Whereas in static culture, BNC production is limited by the restricted air surface area, in the agitated culture, the distribution of oxygen and nutrients in liquid medium is more homogeneous, and therefore, an improved productivity may be expected [24]. However, some shortcomings are also associated with it. One of the major challenges is the genetic instability related to some BNC producer strains (e.g., from *Komagataeibacter* genus) that under agitation may form spontaneous cellulose-negative (Cel^−^) mutants (viz., non-cellulose producers), thereby lowering the overall productivity [16]. Another factor that may negatively influence the productivity of BNC under agitation is the non-Newtonian behavior of BNC in the fermentation medium that will cause a significant increase in the viscosity with the consequent reduction of homogeneity and distribution of oxygen in the medium [50,51].

With the increasing interest in the commercial exploitation of BNC, there has been a significant investment in research to overcome the limitations of both static and agitated cultures in order to achieve high production rates, reduced production costs, and/or shorter cultivation times [44]. The design and development of efficient bioreactors has been one of the explored strategies for the scale-up. In this context, agitated culture bioreactors from the airlift [54] or modified airlift spherical bubble column reactor types [55] are among the ones tested to produce BNC pellets, fibrous material, or sphere-like particles. The production of BNC membranes or pellicles has also been investigated using different bioreactor typologies, including membrane [56], horizontal lift [57], rotating disk [58], or modified airlift [59]. Despite the advances in this field, the use of bioreactors to produce BNC is still limited and is mostly reduced to some tests at the pilot scale. The research done on bioreactors to produce BNC was covered in detail by previous reviews [44,47,48,60].

As in other microbial fermentation processes, the culture medium has a high contribution to the overall cost of BNC production [61]. In addition to the commonly used synthetic culture medium HS, other media have also been investigated and, depending on those, the BNC production cost can be so distinct as ca. €60 to ca. €9000 per kilogram [62]. In this context, over the last few years, as a means to decrease the BNC production costs, several studies have investigated and reported the use of agro-industrial by-products as a fermentation medium with or without specific nutritional supplementation (notably with N and P sources), showing promising results in terms of productivity when compared with the HS medium. Examples include the works using residues of wine and pulp industries [63] or dry olive mill residue water extracts [64] and, more recently, citrus peel and pomace [65], corn steep liquor [52], beverage industrial waste, tomato juice [66], palm date, fig, and sugarcane molasses [67], and cashew apple juice and soybean molasses [68], indicating that this can be a promising approach to reduce the BNC production costs. Finally, the importance of the bacterial strain and its growth characteristics in the global process of BNC production is also well known, and the isolation of more efficient and productive bacterial strains has deserved attention by researchers. However, with the advent of genome sequencing, genetic engineering appears as a more promising approach to obtain more stable and high-yield mutant strains, despite the regulatory issues that some fields may encounter [16,48,69]. BNC production methods were recently addressed in two different reviews [70,71] with discussion about several biotechnological approaches to optimize BNC production [70].

## 3. Properties and Applications of Bacterial Nanocellulose

**Figure 2:**
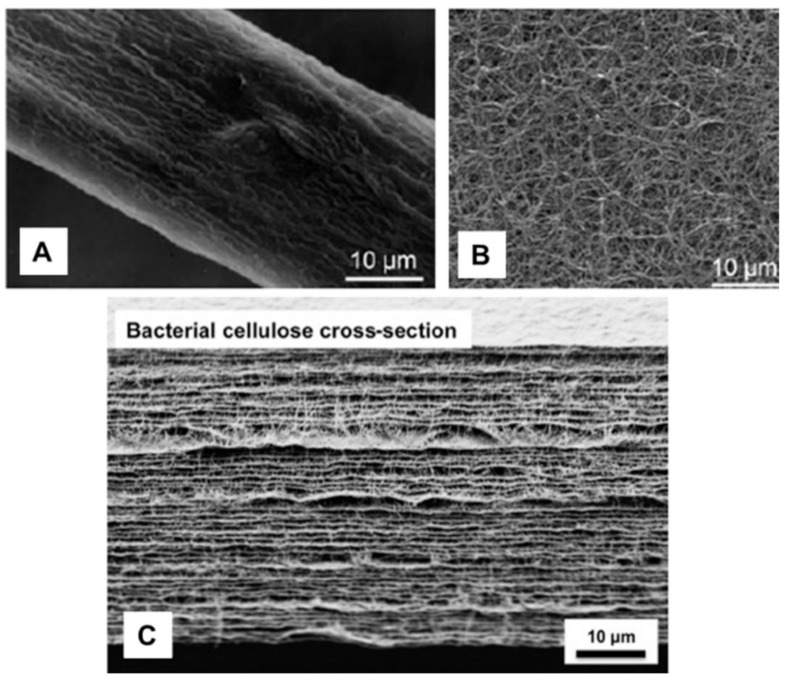
Comparison of electron micrographs of plant cellulose (**A**) and bacterial nanocellulose (BNC) fibers (**B**) (reprinted with permission from [42]); electron micrograph of a cross-section of BNC (**C**) (Reprinted with permission from [29]).

BNC is produced as a well-organized 3D network of ribbon-shaped nano- and microfibrils with extremely reduced diameter when compared with plant cellulose fibers (Figure 2A,B) [42]. An electron microscopy cross-section image of a BNC membrane (Figure 2C) shows that BNC fibers are organized as stratified layers, bundled one after the other as the bacteria grow at the culture medium–air interface [29]. Another property of BNC that stands out over plant cellulose is its high chemical purity, being free of lignin, hemicelluloses, and pectin, typically present in plant fibers [14]. This eliminates the need for complex purification procedures, which are energy and chemically demanding, with reduced yields that may affect the structure and physicochemical properties of cellulose [72].

BNC substrate is characterized by a highly porous nanofibrillar structure, with pore diameters typically below 10 μm, that results in notable permeability to gases and liquids and a very high water-binding capacity (water content >90%) [26,42,73]. It is also characterized by a high crystallinity index (80–90%) that contributes to the high thermal stability of BNC, which is an important characteristic when thermal sterilization is required [36]. BNC shows a high degree of polymerization, typically between 4000 and 10,000 anhydro-glucose units [42]. Due to its crystalline nanofibrillar structure, BNC also shows excellent mechanical properties. Despite some disparity in the literature data, it is generally assumed to have a tensile strength between 200 and 300 MPa and a Young’s modulus reaching values as high as 15–35 GPa [36].

In addition to the remarkable physicochemical and mechanical properties, BNC presents also high in vivo skin biocompatibility, as demonstrated in studies performed with human volunteers [74,75], which is a particular attractive feature when intended to be applied in skin-care applications.

**Figure 3:**
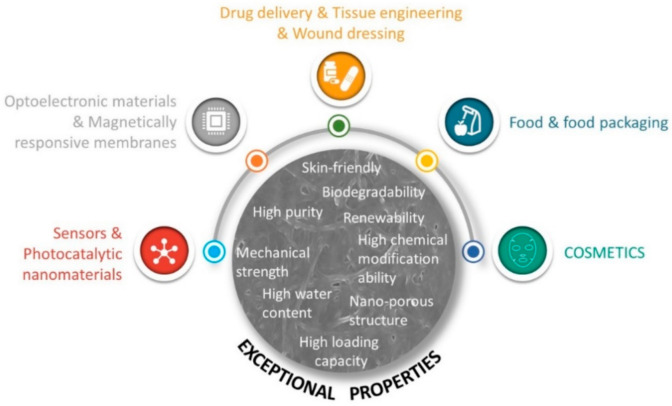
General properties and applications of bacterial nanocellulose.

In addition to shape, the supramolecular structure of BNC, as well as its physical and mechanical properties can be modulated during biosynthesis by altering conditions, such as the production method [44], carbon source [76], bacteria strain [77], or even by adding additives to the culture medium [78], this being a great advantage over plant cellulose. Therefore, these parameters should be carefully selected and controlled considering the desired properties and intended application. In addition, as any cellulose substrate, BNC is also prone to be modified ex situ (after purification), either by chemical treatment (e.g., acetylation, alkylation, carboxymethylation, among others) or by the incorporation of other materials into the BNC network [79,80]. The high surface area of BNC fibrils contributes to the establishment of interactions between the abundant hydroxyl groups in the surface of BNC and functional groups of other components, such as active low-molecular-weight compounds [75,81], polymers [82,83,84,85], metal oxides, or metal nanoparticles [86,87,88,89]. The combination with other materials imparts novel or improved properties to the pristine BNC, such as bioactivity (e.g., antioxidant, anti-inflammatory, and antimicrobial activities), conductive, optical, or magnetic properties or even improved mechanical properties, that greatly extends the potential of BNC and allows designing high-value functional BNC-based materials with application in several fields [79]. The progress done in the development of functionalized BNC-based materials has been addressed in several reviews covering the strategies applied to enhance BNC properties that are valued in areas such as sensors, photocatalytic nanomaterials, optoelectronic materials and devices, magnetically responsive membranes [79], drug delivery [21,22], tissue engineering [80,90], wound dressing [26], or food and food packaging [20]. The functionalization of BNC through chemical modification or in combination with other polymers or active compounds has also been a successful strategy for the exploitation of BNC in cosmetics, as will be discussed in the next section. The properties and main applications of BNC are summarized in Figure 3.

## 4. Application of Bacterial Nanocellulose in Cosmetics

**Figure 4:**
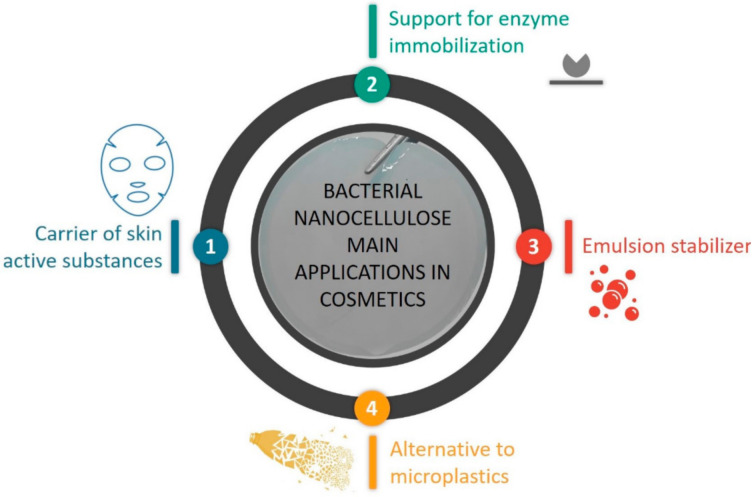
Main applications of bacterial nanocellulose in cosmetics.

Although there are slight variations in cosmetic definition among the three main trading blocks of the cosmetic market, viz., Europe, USA, and Japan, a cosmetic can be generally defined as a product used to improve the appearance of the human body without affecting its basic structure [91]. More recently, another concept was introduced, the cosmeceuticals, which are categorized as products associating features of cosmetics and pharmaceuticals. They incorporate active substances with therapeutic benefits and are applied to treat the most skin-relevant conditions such as photoaging, wrinkles, skin dehydration, dark spots, or hyperpigmentation [91,92]. BNC encounters several potential applications in cosmetics, due to its exceptional properties as referred above, mainly as a support for skin-active substances or as a structuring agent in cosmetic formulations. Table 1 summarizes the most recent (last 5 years) and relevant developments regarding the application of BNC or BNC-based materials in cosmetics, which are achieved in both academic and industrial contexts. The subsequent sections will highlight the use of BNC in cosmetics, notably as a carrier of skin active substances, enzyme immobilizer, emulsion stabilizer, or as an alternative to microplastics, aiming to demonstrate the enormous potential of this remarkable biopolymer as a sustainable alternative for the design of green cosmetics (Figure 4).

### 4.1. Bacterial Nanocellulose as Carrier of Skin Active Substances

BNC has attracted much attention as a delivery system because of its peculiar porous nanostructure that favors the incorporation and release of active substances with interest for areas such as biomedical or cosmetic [93,94]. The successful application of BNC in the transdermal drug release of active substances has been widely demonstrated for both hydrophobic and hydrophilic drugs [22], but the number of studies addressing the BNC release of skin-active substances in cosmeceuticals is more limited. However, over the last years, the suitability and applicability of BNC as the support material of sheet facial masks enriched with active substances, taking advantage of its high mechanical performance, loading capacity, and membrane-like shape, have been increasingly investigated.

Sheet facial masks are typically made of a diversity of fabrics (e.g., cotton, non-woven) and synthetic polymers (e.g., poly (vinyl alcohol) (PVA)) and are currently one of the most popular and fast-growing types of facial products, thus representing an important and highly competitive niche of the cosmetic market [95,96]. The popularity of facial sheet masks is largely due to its easy application and removal, fast use, and effective results that make them very appealing for the nowadays consumers, compared with other mask types (e.g., rinse-off or peel-out) [97]. The mechanical strength and elasticity of BNC (in the wet state) make it particularly resistant to handle, easy to use, and enable a perfect adhesion to skin, enhancing the penetration of active substances into the skin. All these aspects make BNC an exceptional bio-based material for this application and, in fact, some sheet masks made of BNC are already commercially available, such as the CELMAT^®^ (BOWIL—Biotech Ltd, Wladyslawowo, Poland), Intense Whitening Biocellulose Mask (Elizabeth Arden, New York, NY, USA), DHC Bio Cellulose Mask (DHC, Tokyo, Japan), Blank Expert Second Whitening Bio-Cellulose Mask (Lancôme, Paris, France), or Second Skin Mask with Aloe vera (CORYMER, Neerwinden, Belgium) [33], just to enumerate some examples.

In the production of BNC-based facial masks, several works have adopted a bottom–up approach to incorporate the skin-active substances, in particular, natural compounds, into the BNC membrane by adding them to the culture medium, thus promoting their incorporation within the BNC fibril network during biosynthesis, which in theory turns the scale-up easier and may reduce the costs. This was the approach used in the method claimed in a patent by Ho et al. [98] to produce BNC facial masks, in which BNC was biosynthesized in a culture medium containing a bamboo extract (0.01–100%, *w*/*w*), which may be supplemented with additional nitrogen and carbon sources to increase the BNC yield. After production, the BNC membrane was purified by alkaline treatment, sterilized (ideally by hydrothermal sterilization), and dried. The presence of phenolic and carbonyl compounds from bamboo extract was confirmed in BNC membranes after biosynthesis. However, it is not clear if this evaluation was done before and/or after the purification and sterilization. Furthermore, the BNC facial mask was soaked in a cosmetic emulsion and combined with a facial scrub (particles selected from the group consisting of beads, including jojoba esters, alginate beads, and mixtures thereof in rose powder). The bamboo extract (rich in flavonoids, lactones, and phenolic acids, among others) is known to have antioxidant, antiallergic, and skin-whitening properties [99,100]. Tests with volunteers showed a superior performance in terms of adhesion, skin elasticity, softness, and moisturizing effect of the obtained BNC–bamboo extract facial mask comparing with a commercially available rayon non-woven fabric-based mask impregnated with the same cosmetic emulsion [98]. A method to produce BNC using *Moringa oleifera* leaves powder as the nitrogen source in the presence of a protease and ethanol was also claimed in a recent patent [101]. Moreover, after BNC biosynthesis, the resulting fermentation broth, containing enzymatically hydrolyzed proteins, flavonoids, polyphenols, and organic acids from the *M. oleifera* leaves, was recovered and used to prepare a cosmetic formulation that was embedded in the biosynthesized BNC membrane. Results in volunteers showed that the BNC membrane with the *M. oleifera* fermentation broth-based formulation had a better hydration effect than a normal membrane cloth loaded with the same cosmetic formulation.

With modern consumers demanding highly efficient skin care products, the cosmetic industry has been investing in the development of formulations, including facial masks with enhanced skin penetration and absorption [102]. In this context, Gregoire et al. [103] developed a patented method to prepare a composite sheet with a multilayer structure composed by BNC biosynthesized in the presence of *Sphaerotilus natans*-derived microtubes (composed by a polysaccharide, commonly a deoxy sugar-modified (β1→4)-linked glycosaminoglycan). Permeation assays using a synthetic membrane equivalent to skin showed that the obtained BNC–microtubes composite sheet had an improved transdermal delivery of cosmetic actives (caffein, minoxidil, 3-(2,4-dihydroxybenzyl-1-2-(2-hydroxyethyl)-pyrrolidine-2,5-dione) and 4-(tetrahydro-pyran-4-yl)benzene-1,3-diol, oxybenzone) compared with a BNC sheet without microtubes. This result was valid for both water-soluble and lipid-soluble active ingredients [103].

Over the years, and in an attempt to reduce the costs of BNC production, several works have been adopting the strategy of using food and agroforest by-products of natural origin to biosynthesize BNC for cosmetic applications. An example of this is the method claimed in a patent to produce BNC matrices using soybean molasses [104] as the nitrogen and/or carbon sources of the BNC fermentation broth. The BNC matrix obtained with soybean molasses substrate and immersed in a cosmetic essence with glycerin, polyethylene glycol, and carbomer was shown to have a high-water retention rate (98.35%) and good moisturizing effect [104]. The authors claimed thereby that the soybean molasses can be considered as a viable alternative to the standard HS medium for the production of BNC matrices for facial masks, with economic and environmental profits. In another patent, a method to produce BNC using expired milk or its derivatives (0.5–3.0% *w*/*v*) as a carbon source was described [105]. In addition to expired milk, tea polyphenols (0.01–0.10% *w*/*v*) were also added to the culture medium to confer functional properties to the BNC membrane. The use of milk and tea polyphenols resulted in an increase in the BNC production yield when compared with glucose-based medium. Apparently, the content of tea polyphenols was not determined in the BNC membranes after being purified by alkaline treatment and subsequent washing until neutrality; however, the BNC membranes demonstrated antioxidant and whitening properties [105], indicating that these bioactive compounds were incorporated into BNC. The production of BNC by *K. hansenii* in a culture medium containing 13% of tropical fruit by-products, known for having considerable amounts of vitamins and minerals with recognized benefits for skin, was also reported by Amorim et al. [106]. The results showed that the obtained BNC membrane presented good mechanical performance and a high-water activity (99.13 ± 0.09%). The authors also confirmed the incorporation of ascorbic acid from the fruit residues (24.4 ± 1.01 mg per 100 g cellulose) using the Tillmans method. The incorporation of this active substance into BNC makes possible its use in cosmetic as a facial mask with hydrating and antioxidant actions [106]; however, this was not demonstrated in the scope of the study.

The in situ incorporation of active substances into BNC by adding the potential bioactive compounds to culture medium, although appealing in terms of scale-up, has to take into consideration some important issues, namely, the fact that the BNC purification process, after biosynthesis, has several washing steps with water and a treatment with an alkaline solution, which may cause significant losses of water-soluble bioactive compounds. Thus, to ensure the efficacy of the method, the effective incorporation of these compounds should be carefully assessed by performing the structural characterization of the purified BNC matrix. However, this evaluation is not always clearly shown, as was highlighted in some of the studies/patents described above.

Alternatively, regarding the in situ incorporation approach, the pure BNC or BNC-based composite membranes can be soaked in a solution of the active substances of interest, which in fact is the most commonly adopted method for the production of functional BNC materials for cosmetic applications. This was the strategy used by Silva et al. [107] to investigate the potential of BNC membranes as a topical delivery system of caffein for cellulite treatment. Scanning electron microscopy (SEM) micrographs showed that the obtained BNC–caffein (caffein content of 8 mg cm^−2^) membranes were homogeneous with no agglomerates’ formation. In addition, the in vitro permeation studies using epidermal membranes demonstrated that BNC–caffein membranes had lower permeation rates than the ones obtained with conventional formulations (aqueous solution and gel) also included in the assay. Specifically, BNC–caffein membranes presented a caffein flux of 2.55 μg cm^−2^ h^−1^ and a cumulative amount permeated after 10 h of 0.39% (percentage of applied dose), whereas the aqueous solution provided the highest flux (7.53 μg cm^−2^ h^−1^) and percentage of caffein permeated (0.97%). As the authors point out, the complex 3D network of BNC makes probably the diffusion pathway of caffein tortuous and might be responsible for the slow release of caffein from BNC, which is desired for this type of formulation whose effect is expected to persist over time. In addition, the use of this BNC–caffein system also overcomes the lack of reproducibility of conventional gel formulas regarding the dose of caffein that it is applied and losses caused by absorption due to contact with clothing or other surfaces [107].

Numata et al. [108] have also followed a simple approach of BNC incorporation to design a cosmetic delivery system, combining a BNC gel with poly(ethylene oxide)-*b*-poly(caprolactone) (PEO-*b*-PCL) nanoparticles by diffusion of the nanoparticles into the gel. To assess the effectiveness of the delivery system, PEO-*b*-PCL nanoparticles were loaded with retinol, a hydrophobic molecule, which is widely used as an anti-aging ingredient. The releasing assays performed in acetic acid–sodium acetate buffer (pH 5.2) showed that retinol is slowly released from PEO-*b*-PCL nanoparticles inside the BNC membrane; however, the results also revealed that most of the retinol released from nanoparticles precipitated and stayed retained in the BNC gel [108]. Despite the need for further studies to overcome this problem, these findings are a good starting point to develop BNC-based delivery systems for hydrophobic active substances that, owing to the intrinsic hydrophilic nature of BNC, is still a challenging issue.

**Figure 5:**
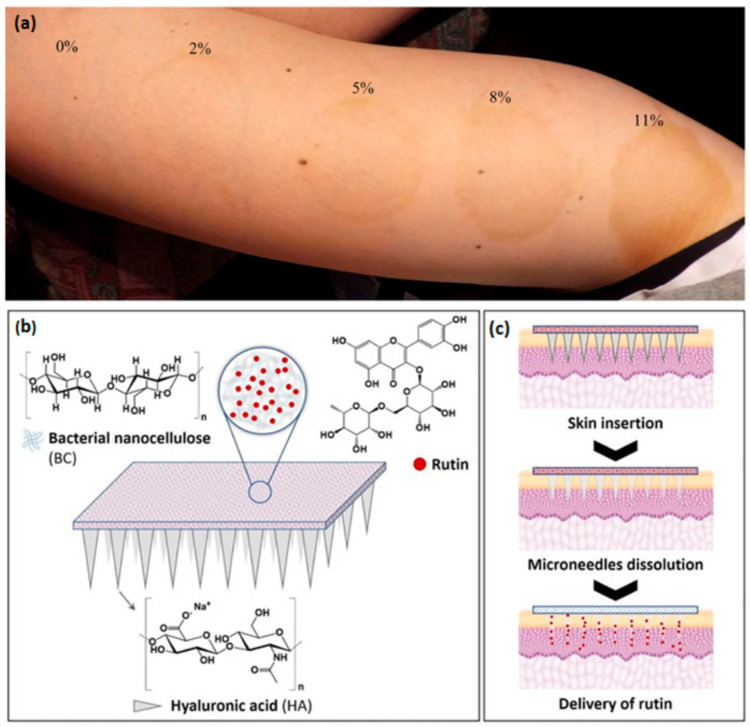
Skin coloring 12 h after removal of BNC–DHA patches (concentration of DHA is expressed in percent), applied for 30 min (**a**) (reprinted with permission from [110]); Schematic representation of the HA-(BNC-R) MNs structure (**b**) and functioning of this innovative system: insertion of the MNs into the skin, dissolution of HA MNs and subsequent release of the bioactive molecule from the BNC membrane (**c**) (reprinted with permission from [119]). DHA: 1,3-dihydroxy-2-propanone, HA: hyaluronic acid, MN: microneedles, R: rutin.

In the same vein, a method to produce a BNC mask with anti-inflammatory and anti-allergic properties incorporating bee venom (rich in melittin) was claimed in a patent by Hongli et al. [109]. In this study, BNC was used not only as the support of the bee venom active components but also as emulsifier and thickener, in the form of nanofiber filaments, of the water-soluble bee venom, replacing the traditional chemical thickeners used in these formulations [109]. The use of BNC membranes as a carrier was also evaluated for 1,3-dihydroxy-2-propanone (DHA), which is widely used in cosmetics as an active ingredient in self-tanning products [110]. BNC membranes were impregnated with different DHA concentrations and afterwards tested on skin. The findings clearly indicated that after 30 min of application, the skin pigmentation increased with the DHA concentration (Figure 5a). The pigmentation of skin by DHA is based on a typical Maillard reaction (viz., reaction of sugars with amino acids) in which DHA reacts with keratin on the skin surface, forming pigments (melanoidins) bounded to the proteins of the *stratum corneum* through lysine chains. This is a reaction limited only to this outer layer of the skin [111]. Noteworthy is also the fact that the BNC–DHA patches did not leave the specific and typical unpleasant odor of cosmetics containing DHA, which only disappears with the skin pigmentation effect. As concluded, the method requires improvement, but it demonstrated the potentiality of BNC as a carrier of DHA with the advantage of having the ability of being shaped accordingly to the skin surface on which it will be applied. Therefore, this work opens the possibility of BNC being applied in personalized patches to mask vitiligo symptoms or in self-tanning masks that may be enriched with other active ingredients (e.g., anti-aging compounds) [110]. In another study, Pacheco et al. [96] also demonstrated the potential of BNC as a sustainable alternative vehicle for cosmetic actives by investigating the incorporation of two formulations into BNC: the vegetable extract mask (VEM) consisting of a moisturizing formulation based on Hidroviton^®^ (moisturizing agent) and plant extracts (oat and rosemary) and the other composed by a propolis extract and poly(propylene glycol) (PEG), which is identified as a propolis extract mask (PEM). Hidroviton^®^ and PEG acted as plasticizers, giving flexibility to the membranes. SEM micrographs confirmed the incorporation of the cosmetic actives into the BNC network without affecting its morphology. The release assays in phosphate-buffered saline (PBS) were followed by infrared spectroscopy, showing that the skin-active substances were quickly released from BNC, which is particularly relevant for a facial mask whose application is ideal for a short period of time. The effectiveness and acceptance of BNC membranes were evaluated through sensory tests in volunteers, which revealed high scores in skin adhesion and mask handling because of the improved malleability given by the incorporated formulations. The effect of the active substances on skin moisture was also evaluated, resulting in an increased hydration effect for VEM treatment and a decrease on skin hydration for PEM formulation after a 20 min use per day during 5 consecutive days [96]. In another recent study, Amorim et al. [112] investigated the incorporation of 2% of aqueous propolis extract in BNC membranes simply by immersion in the extract solution. The propolis extract is commonly used in dermatological preparations for wound healing and treatment of burns and acne [113]. Thus, the aim of the authors was to develop a material to be used as a facial mask for the treatment and healing of acne-prone skin. The results showed that with the incorporation of propolis, the BNC crystallinity was reduced, which directly influences the mechanical properties of the membrane, resulting in a more elastic material with improved flexibility. This increased flexibility is favorable to a better adhesion and adaptation of a facial mask to skin, demonstrating therefore the potential applicability of the obtained BNC–propolis membrane for this purpose [112].

Following the trend of BNC skin masks with improved properties and delivery of active ingredients, researchers developed innovative bioactive BNC membranes by loading with ionic liquids (ILs) based on phenolic acids [114]. Specifically, cholinium cation was combined with anions derived from ellagic ([Chol]_2_[Ell]), caffeic ([Chol][Caf]), and gallic acids ([Chol][Gal]), which are natural antioxidants used in cosmetics. The synthesis of phenolic-based ILs, viz., solvents with a green connotation, aimed to improve the antioxidant activity of these phenolic compounds and at the same time overcome the problems of the low solubility and bioavailability commonly associated with phenolic compounds. The bioactive BNC-ILs membranes showed higher re-hydration ability, which is important to assure suitable hydration for the ILs release. The in vitro skin permeation assays (during 5 h), using human epidermal skin in Hanson vertical diffusion cells and PBS buffer at 37 °C, revealed that the bioactive BNC-ILs membranes exhibited a slow and sustained release of the active compounds with a higher flux for BNC-[Chol][Gal] (5.42 μg cm^−2^ h^−1^) than for the BNC-[Chol][Caf] (4.93 μg cm^−2^ h^−1^). The permeation of BNC-[Chol]_2_[Ell] was not evaluated due to precipitation problems. The BNC-ILs membranes were also non-cytotoxic and had high antioxidant and anti-inflammatory activities as desired [114]. Although the slow release of active compounds is not ideal for a facial mask, it may be interesting for other dermal care applications. For instance, for membranes loaded with the anions derived from caffeic acid, BNC-[Chol][Caf] may be used as cellulite treatment patches, where the continuous and controlled release is required. In the same research line, another study reported the incorporation of vitamin B-based ILs ([Chol][VITS]) in BNC membranes [115]. Here, the cholinium cation was paired with anions derived from vitamins of complex B commonly used in skincare formulations, namely, nicotinate (B3), pantothenate (B5), and pyridoxylate (B6). The resulting membranes (dried state) had reduced brittleness due to the plasticizer effect of the ILs, thus avoiding the use of additional plasticizers. Moreover, BNC-ILs membranes had an improved re-hydration ability (from 2.9 to 4.8-fold) in comparison with BNC. Dissolution tests in PBS showed a fast release of 66% of the incorporated [Chol][VITS] from the corresponding membranes in the first 5 min, which, as authors discussed, is particularly attractive for short-term masks. Moreover, the membranes showed to be non-cytotoxic to skin epithelial cells, reinforcing the suitability of these membranes for skincare applications. Therefore, future studies should address biological assays regarding the anti-aging properties of ILs such as the collagen production or the enhancement moisturizing of the *stratum corneum* skin layer [115].

BNC can also be combined in different ways with bioactive macromolecules. Hyaluronic acid (HA) is a main constituent of skin epidermis and dermis with recognized properties in skin moisture maintenance and skin-aging process [116], and thus is a crucial ingredient for cosmetics, including BNC-based masks. Examples are the methods claimed in two patents to prepare nanocomposite facial masks of BNC and HA [117] and sericin–HA [118], which are both prepared by addition of the active molecules to the culture medium. More recently, an innovative patch for dermo-cosmetic applications (e.g., skin anti-aging) was developed by Fonseca et al. [119] using the non-invasive technology of microneedles (MNs). For further reading about this technology, a recent review gives a comprehensive overview about the last developments on polysaccharides and protein-based MNs [120]. The system developed by Fonseca et al. [119] combines the features of HA as the dissolvable MN matrix to enable improvement of the overall appearance of the skin, with the properties of BNC as the back layer to support the MN array and enable the release of additional active molecules (Figure 5b,c) [119]. The effectiveness of the system was demonstrated with the incorporation of the bioactive compound rutin, a natural antioxidant, in the BNC back layer of the MNs system. Results showed that the incorporation in the MNs system did not affect the antioxidant activity of rutin, and this activity was maintained for 6 months storage at room temperature. Additionally, the in vitro skin assays also unveiled the successful penetration of these arrays through the skin and the delivery of rutin. The safety and cutaneous compatibility of the MNs HA–BNC were also evaluated and demonstrated in a preliminary in vivo assay performed in human volunteers, in which no significant changes in barrier function, *stratum corneum* hydration, nor redness were detected [119]. This work reinforces the potential of BNC as a delivery system in cosmetics.

Still in the field of facial masks, a recent innovative approach was used to improve the delivery of cosmetic actives in a sheet (e.g., carbon cloth, cotton) facial mask by including a battery [121]. The authors used a BNC carbide sheet as the positive electrode, which was obtained by carbonization of the BNC xerogel at 1200 °C under nitrogen atmosphere. The negative electrode contained at least one of these elements: magnesium, zinc, aluminum, iron, calcium, lithium, or sodium. Since a facial mask is a disposable product, the aim was to develop a battery using a material with low environmental impact. The authors claim that the weak electric current flow generated by the battery allows the active ingredients to penetrate more effectively into tissues and at the same time enable an electrical stimulation of face muscles and lymph. The evaluation tests on volunteers confirmed that the battery-sheet facial mask, impregnated with active ingredients (aqueous solution of carbonic and L-ascorbic acids), had effectively increased skin moisture content when compared with a sheet facial mask impregnated with the same active ingredients but without a battery.

The development of BNC-based materials with improved properties whether mechanical, viscoelastic, or swelling behavior that favor their performance in cosmetics has also been the aim of a few works. An example is the research describing the development of a nanocomposite gel composed of BNC–poly(ethylene glycol diacrylate) (PEGDA) aiming to obtain an improved soft and flexible gel material either for cosmetic or biomedical applications [122]. A series of compositions were investigated varying the ratio of PEGDA in the BNC–PEGDA gels. Results showed that the BNC gels with 3% (*w*/*v*) and 5% (*w*/*v*) of PEGDA were the ones with higher potential for this type of application, considering their mechanical and viscoelastic properties. These BNC–PEGDA gels revealed to be harder (deformation resistance against compression) but less brittle against tension than a pure BNC gel. The hardness prevents the change in tactile sensation after repeated finger contacts that in the case of BNC lead to water expelling and consequent dryness of the gel. Additionally, results also demonstrated that BNC–PEGDA gels had a similar viscoelastic behavior to that of BNC gel, with elastic properties being more significant than viscous ones. This indicates that the low amounts of PEGDA did not significantly affect these properties and these composite gels could adhere to skin. Although no in vivo tests have been performed, this nanocomposite is believed to have good biocompatibility, considering that both polymers are biocompatible, what is also favorable for its use in cosmetic or medical fields [122], but further studies should be performed to fully prove the applicability of these BNC–PEGDA gels as, for instance, a support matrix for delivery of cosmetic actives.

In another study, Chunshom et al. [123] successfully prepared a freeze-dried BNC/PVA nanocomposite in which BNC was used as a reinforcement material to benefit from its mechanical and thermal properties. The blend of BNC and PVA has been the focus of several previous works specially for biomedical applications [124]. However, in this study, the innovative approach of producing a freeze-dried BNC/PVA nanocomposite makes it attractive not only for biomedical but also for personal care cosmetics, as the dried-state products are nowadays gaining relevance in products such as cleansing masks and moisturizers [123]. One advantage of a dried-state nanocomposite is the reduced possibility of microbial contamination compared with a never-dried hydrogel counterpart in which the water in the 3D network can favor it. Moreover, its lightweight is also an attractive property for industrial application, since it facilitates packaging, transportation and storage. The results revealed that BNC had a significant influence on the composite pore size (10 μm–100 nm), which is likely contributing to the swelling behavior of the nanocomposite gel. Several composites with different weight ratios of PVA:BNC were studied. The composite gel with a ratio of 3:1 (PVA:BNC) presented an excellent swelling behavior within 30 min either in deionized water, NaCl solution, or PBS [123], which is a promising result for its application as a hydrogel in short-term cosmetic applications, although further studies need to be conducted.

An essential issue in the manufacturing of cosmetic formulations is to assure their quality and safety, including stability over time. Regarding BNC cosmetic masks, this aspect is not often covered in the literature. However, a novel and simple methodological approach that can be applied in future studies was recently proposed by Perugini et al. [93]. The authors investigated the suitability of two non-destructive instrumental techniques, namely, near-infrared spectroscopy (NIR) and multiple light scattering (MLS), for the quality assessment of BNC facial masks. In particular, the NIR technique was used to perform a quality check by evaluating homogeneity and reproducibility among masks of the same manufacturing batch and from different batches with regard to water and ingredients distribution. On the other hand, MLS was applied to ascertain the stability of the cosmetic masks and the possible interaction between the formulation and the BNC matrix, which are important criteria to establish the shelf-life of these products [93]. Another aspect that is not always included in the development of new BNC facial masks is their acceptance and effectiveness using in vivo assays. In this perspective, Perugini et al. [97] recently provided a scientific protocol to evaluate, in vivo, the efficacy of topical treatments with BNC masks employing a methodology that combines non-invasive skin bioengineering techniques and statistical methods. The authors evaluated the skin effect of three BNC facial masks embedded with cosmetic formulations with different beneficial skin actions (anti-aging, lifting, and cell renewal). The study was performed in volunteers and evaluated a broad range of skin effects, such as moisturization, color, viscoelastic properties, and surface smoothness of skin, as well as the presence of wrinkles, dermal homogeneity, and *stratum corneum* renewal. The results were very satisfactory for all masks, highlighting the potential of this biopolymer to develop tailored cosmetic masks with high tolerability and efficacy, validating at the same time a non-invasive approach to evaluate the in vivo effectiveness of BNC facial masks [97].

### 4.2. Bacterial Nanocellulose as a Support for the Immobilization of Enzymes

In cosmetics, enzymes have been applied for many years, as is the case of some proteolytic enzymes (bromelain and papain) used for skin peeling and smoothing, or the superoxide dismutase (SOD) known for its capacity in removing free radicals to prevent the associated skin aging [125]. However, despite their potential as skin-active substances, enzymes encounter some problems, such as poor stability at room temperature during prolonged storage or deactivation due to disturbance of the enzyme structure by oils and surfactants commonly present in cosmetics [126]. In this context, the nano-porous structure and high-surface area of BNC make it an attractive substrate for this application, offering benefits in terms of the enzyme entrapment and immobilization yield as well as a better enzyme stabilization [127,128]. In fact, the use of the BNC matrix has already been investigated to immobilize enzymes such as laccase, which is an enzyme used in food and paper industries [129,130], or the horseradish peroxidase, which is applied in biotechnological and environmental applications such as the removal of contaminants from water [127]. However, the use of BNC as a support matrix to immobilize enzymes in cosmetic applications is almost unexplored. In our literature survey, only a recent study performed by Vasconcelos et al. [128] describes the covalent immobilization of papain in wet BNC membranes previously purified by alkaline treatment and oxidized via NaIO_4_. On cellulosic supports, among the possible methods, the covalent bonding promotes the most stable interaction with enzymes, resulting in a possible increase of the activity and thermostability of the immobilized enzyme [128]. However, BNC hydroxyl groups do not react directly with the amine groups of enzymes [127], and thus, a potential solution to assure a more stable interaction is to chemically modify BNC [128]. In this study, covalent bonding was achieved by imine formation between the protein amino groups and the BNC carbonyl groups produced by an oxidative treatment. It was observed that the percentage of recovered activity of the enzyme (relates the activity of immobilized versus free enzyme) after immobilization on the oxidized membranes was 93.1%, confirming that the papain remains active after immobilization. Moreover, compared with non-oxidized BNC, a higher amount of enzyme was immobilized on the oxidized membrane because of a better chemical interaction with the enzyme, owing to the covalent imine bond established between the aldehyde groups (oxidation result) of BNC and the amine groups of the amino acids structure from the enzyme, as evident by infrared spectroscopy [128]. The thermogravimetric analysis showed that the oxidation reaction had lowered (about 54°C), the onset temperature (T_Onset_) of the BNC membrane, indicating a reduction in its thermal stability. However, the T_Onset_ of 262 °C for the oxidized membrane is still sufficiently high for autoclaving. Finally, the oxidation reaction had also resulted in BNC membranes with reduced mechanical resistance and higher flexibility, showing a Young’s modulus of 1.7 ± 0.18 MPa (non-oxidized BNC: 5.3 ± 0.79 MPa), a tensile strength of 0.3 ± 0.02 MPa (non-oxidized BNC: 1.1 ± 0.05 MPa), and an elongation at break of 31.0 ± 3.55% (non-oxidized BNC: 26.6 ± 1.28%). However, from our perspective, the mechanical resistance is still adequate for cosmetic applications, and the increased flexibility is beneficial for the adherence to skin. Although this study is not specifically oriented to the cosmetic domain, it demonstrates the potential of the method to oxidize the wet BNC and to use it as a support for the immobilization of a cosmetic enzyme, despite further proof-of-concept studies needed to fully prove it. Therefore, given the growth prospect for the use of enzymes in cosmetics, owing to their natural origin, consumer acceptance, and high performance, this must be a research field in which BNC may gain relevance in the next years in a viewpoint of a sustainable and eco-friendly products development.

### 4.3. Bacterial Nanocellulose as Emulsion Stabilizer

**Figure 6:**
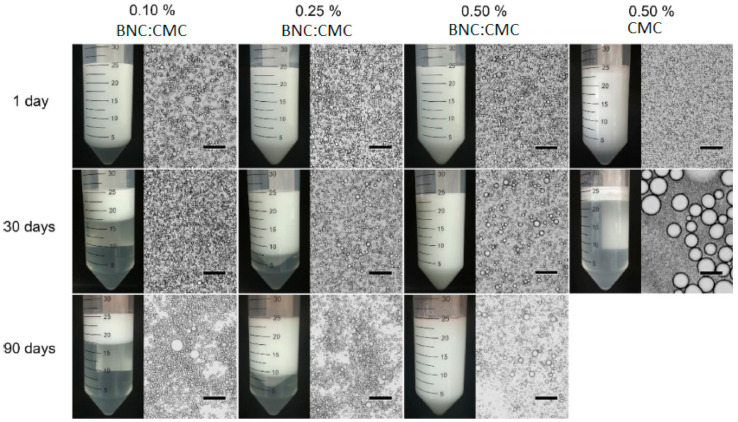
Digital photographs and optical micrographs (10× magnification) of 10% isohexadecane-in-water emulsions prepared with different concentrations of BNC:CMC dry formulation (0.10%, 0.25%, and 0.50%) and with 0.50% CMC, taken 1 day after preparation and 30 and 90 days after storage at room temperature. Black scale bars correspond to 100 μm (reprinted with permission from [134]). CMC: carboxymethyl cellulose.

An emulsion consists of a heterogeneous mixture of two immiscible liquids in which droplets of one are dispersed on the other, being typically stabilized by surfactants [131]. Over the last few years, benefiting from advances in nanotechnology and due to irritating, toxic, and environmental problems generally associated with synthetic surfactants, Pickering emulsions (viz., emulsions stabilized by solid particles) have gained commercial relevance over the surfactant-stabilized emulsions [132]. In particular, the use of biopolymeric particles instead of their synthetic counterparts is now emerging as a sustainable and green alternative [133]. In this vein, Jia et al. [132] have produced individualized cellulose nanofibers by the oxidation of dispersed BNC using a mixed system with 2,2,6,6-tetramethylpiperidine 1-oxyl (TEMPO), NaBr, and NaClO, aiming to get a safe, biodegradable, and sustainable solid emulsion stabilizer. The prepared TEMPO-oxidized BNC nanofibrils (TOBNC) showed reduced fibril size and strengthened wettability (accessed by contact angle measurements). Both features were influenced by the degree of oxidation that had increased with the NaClO dosage. The suitable counterbalance between wettability and fibril size seems essential to achieve emulsion stability. Taking in account the results, the authors considered that the TOBNC, prepared with 2 mmol g^–1^ of NaClO, was the most effective emulsion stabilizer against creaming and coalescence, showing a better stabilizing performance than BNC in oil–water type Pickering emulsions (prepared using liquid paraffin) stored at room temperature over 8 months [132]. This is likely due to the fact that in TOBNC, the wettability did not change much, but the fibril size was significantly reduced, resulting then in more fibrils covering the surface of droplets, thus creating a rigid barrier. Given the promising results, the authors foresee the use of this TOBNC as an emulsion stabilizer in topical, pharmaceutical, or food formulations [132]. More recently, another study proposed a one-pot method to produce TOBNC using TEMPO immobilized on silica beads and performing a simple filtration after the oxidation reaction, instead of the centrifugation used in the previous method [12]. This approach ensured the total removal of reactants, which is particularly important for skin care products. Moreover, it was also observed that the obtained TOBNC maintained the nanofibrous structure of BNC and the water-absorbing capacity. When combined with water and an oil–water emulsion, it showed the capacity to block the adhesion of particulate matter on skin using a porcine skin model. By the simplicity, efficiency, and easy scale-up, this one-pot method was shown to be a good alternative to produce TOBNC for skincare applications at the industrial level [12]. Envisioning the industrial application of BNC in cosmetic and food sectors, Martins et al. [134] developed a dry formulation of BNC and carboxymethyl cellulose (CMC) using the spray-drying technique. This novel dry formulation of BNC:CMC demonstrated to be fully dispersible in water within a few minutes using low-energy stirring, which might have a relevant impact in production costs. When compared with commercially available dry celluloses, the dry BNC:CMC formulation had a better stabilizing performance, showing at a concentration of 0.50% the capability of stabilizing low density oil-in-water emulsions against coalescence or creaming for up to 90 days (Figure 6), which is a promising result for its applicability in cosmetics as a Pickering emulsions stabilizer.

### 4.4. Bacterial Nanocellulose as an Alternative to Microplastics in Cosmetics

The environmental impact of microplastics (or plastic microbeads) (<5 mm) is nowadays of major concern, being the cosmetics and personal care products, one of the relevant sources of primary microplastics (viz., manufactured microplastics) [135]. A broad range of daily-use personal care and cosmetic products (e.g., shampoos, toothpastes, facial scrubs, and soaps) contains microplastics that have either a decorative or exfoliating function. Polyethylene is the main polymer used in microplastics production, but other synthetic polymers are also used (e.g., polypropylene, nylon, polytetrafluoroethylene) [136,137]. The most claimed problem of these microplastics is related with their recalcitrancy and with the fact that they ultimately end up in the environment, and in the marine environment in particular, and may build up in the food chain, ultimately causing adverse effects on human health [138]. Thus, as in other fields, over the last few years, the awareness of consumers and the environmental policies are driving the research and industrial players to find environmentally friendly alternatives to microplastics. With this regard, Joonwon [139] developed a patented method to prepare a BNC powder with properties that turn it into a potential and appealing alternative to replace microplastics in cosmetic products (e.g., scrubs, lotions). The method includes the production of BNC sheets (thickness of 7 to 15 mm) in culture medium based on coconut extract and, for example, fruit juice, followed by treatment with an alkaline solution to remove oils and impurities, a step of air drying (60–99 °C), and finally, the pulverization of BNC. The authors claim that the resulting BNC powder, with a minimum purity of 95%, should have a size between 0.1 and 1 mm and a maximum residual water content of 15%. The reduced water content is essential for the BNC powder to have little or no water absorption ability and therefore preserve the shape even in a liquid phase, which is important to maintain granularity. This will be related to the complete collapse of the 3D structure of BNC during the drying process, which prevents BNC from rehydrating. The behavior of the obtained BNC powder was tested in purified water and compared with a conventional dried BNC powder, which confirmed the water non-absorption property of this novel BNC powder [139]. Apparently, no stability tests were performed under this study, but the properties of the BNC powder look promising for it to be applied as a substitute of microplastics.

## 5. Conclusions and Future Perspectives

Over the last years, with the growing demand for bio-based cosmetics, the extraordinary properties of bacterial nanocellulose, along with its biodegradable nature, renewable character, and unique properties, have drawn an increasing attention by both academia and the cosmetic industry. This review makes evident that this remarkable biopolymer is a feasible alternative to petroleum-based counterparts for a broad range of cosmetic applications. Benefiting from its ultrafine nano-porous structure, BNC has been extensively explored as a carrier of skin-active substances, with several studies demonstrating its potential and effectiveness for the loading and releasing of these substances, especially applied as sheet facial masks. In this field, despite the advances and the well accepted products already commercialized by top cosmetic companies, there are still some challenges needing to be deeply studied, namely the design of delivery systems of hydrophobic molecules, which is not straightforward owing to BNC hydrophilicity. As demonstrated in this review, the blend of amphiphilic polymers is a promising strategy. In cosmetics, as in drug-delivery, it is desirable to have a precise delivery system tailored according to the intended application, to reduce the loading dose of the active compound and, at same time, guarantee the effectiveness of the treatment. Therefore, this is an aspect that should continue to deserve attention in further investigations by including in vitro and/or in vivo assays and searching for optimized BNC-based delivery systems with efficient releasing rates that can be modulated by physical treatments, chemical modifications, or even association with other polymeric matrices. In this topic, the design of smart hydrogels, namely temperature-responsive hydrogels, are gaining interest in the skin care field as delivery systems with controlled release based on the body temperature stimulus.

BNC has also been successfully explored, although in few studies, as a sustainable alternative to synthetic surfactants used to stabilize Pickering emulsions. However, studies demonstrating the performance of these BNC-based emulsion stabilizers in realistic cosmetic formulations are still missing. Therefore, considering that Pickering emulsions are an emerging choice in topical formulations as a vehicle of skin-active substances, further investigations should include comparative studies with different cosmetic formulations. Moreover, the influence of the BNC-based particles in the release profile of the active substances from the emulsion and their penetration into skin should also be addressed. In this context, novel combinations of BNC nanofibrils with other biopolymers or the use of BNC nanofibrils with different sizes or shapes may be investigated, aiming at obtaining emulsions with improved properties, regarding stability, cosmetic active release, and skin penetration, similar to what has been done with other biopolymers [133].

Over this review, it is also noteworthy that BNC also demonstrated to be a viable alternative to other relevant cosmetic applications, such as support matrix for enzymes immobilization or as alternative to microplastics, although these two research topics are only in a very early stage. Regarding enzymes immobilization, additional studies are needed with regard to the stability of the enzyme activity upon storage, but the technology based on the immobilization of enzymes onto oxidized BNC seems promising and may be an attractive form of using enzyme-based cosmetics. In this way, the low effectiveness and short shelf-life, many times associated with conventional formulations, may be overcome. Future research should also follow the consumers demand for multifunctional products and consider the development of effective systems with multiple immobilized enzymes.

Cellulose substrates are gaining interest as an environmental-friendly alternative to microplastics [140]. In this regard, a recent patent claims one method to produce BNC powder intended to be used as microbeads in cosmetic formulations, showing thereby that BNC may be a promising substrate for this purpose. However, this possibility is still underexplored, and progress is expected for the next years. In this field, one of the main challenges will be to develop simple, scalable, and cost-effective processes capable of producing microbeads with constant and uniform diameters, allowing a close control of the size, shape, and hardness of the microbeads to meet the desired final product properties.

With cosmetic companies setting strict sustainability objectives, the investment in green-tech solutions has been extended to the entire product life cycle, including packaging. In this context, BNC may also draw attention in the future as an environmentally friendly material for cosmetic packaging, as is already the case in the food sector. In our literature survey, no studies were found that focused on this application. Hence, this should be a research topic that will probably evolve in the future, especially in the segment of active packaging. Herein, the development of BNC composites with compounds that may impart BNC with properties highly valued in cosmetic packaging, such as the O_2_ scavenging, antimicrobial, or antioxidant activities, will be particularly attractive.

Although BNC products are already commercialized by a small number of companies, there are still some economic constraints that are hampering its broad application and should continue to drive future research. However, in this review, it is clear that many studies are already considering the reduction of costs and the up-scaling to mass production either by using agro-industrial by-products as feedstocks or by developing simple and easy processes to be implemented at a large scale. The progress in the fermentation systems using bioreactors and in the genetic engineering of BNC-producing bacteria should also be crucial to achieve high production yields and cost-effective BNC, which will make it more competitive in the cosmetic market and thereby extend its use. However, the versatility of BNC is unquestionable, and, for the coming years, it is expected that this high-performance biopolymer will gain even more relevance in the highly innovative cosmetic industry, especially in green cosmetics.

## Figures and Tables

**Table 1:** Summary of the most recent and relevant research articles and patents reporting the application of BNC for cosmetic purposes.

Active Substance/ Co-Former	Main Outcome	Application/Potential Application	Reference	Year
	BNC AS CARRIER OF SKIN ACTIVE SUBSTANCES			
Bamboo extract added to BNC fermentation broth	BNC membrane with superior performance in terms of adhesion, skin elasticity, softness, and moisturizing effect	Sheet facial mask	[98]	2015
*Moringa oleifera* leaves powder added to BNC fermentation broth; *Moringa oleifera* fermentation broth	BNC membrane with embedded *Moringa oleifera* leaves fermentation broth with a better hydration effect than a normal membrane cloth	Sheet facial mask	[101]	2019
*Sphaerotilus natans*-derived microtubes	Multilayer structure with an improved transdermal delivery of water and lipid-soluble active substances	Sheet facial mask	[103]	2018
Soybean molasses added to BNC fermentation broth	BNC membranes with high-water retention rate (98.35%) and good moisturizing effect	Sheet facial mask	[104]	2018
Milk by-products and tea polyphenols added to BNC fermentation broth	Increased BNC production yield; BNC membrane with antioxidant and whitening properties	Sheet facial mask	[105]	2019
Tropical fruit by-products added to BNC fermentation broth	BNC membrane with high-water activity and incorporating ascorbic acid from the fruit by-products	Sheet facial mask	[106]	2019
Caffein	BNC—caffein topical delivery system with lower permeation rates of caffein than conventional formulations (aqueous solution and gel); reproducible, predictable and extended release of caffein over time	Patches for cellulite treatment	[107]	2014
PEO-*b*-PCL nanoparticles encapsulating retinol	BNC-based delivery system of hydrophobic molecules; slow release of retinol from nanoparticles; retinol precipitation and retention in the BNC gel (further studies are needed)	Hydrogel for skin care	[108]	2015
Bee venom	BNC membrane with potential anti-inflammatory and anti-allergic properties	Sheet facial mask	[109]	2017
DHA	BNC–DHA patch applied for 30 min was effective in conferring a skin natural tan effect	Sheet facial mask	[110]	2018
Hidroviton^®^ and plant extracts/PEG and propolis extract	Effectiveness of BNC facial masks as delivery system of active substances	Sheet facial mask	[96]	2018
Propolis extract	Improved flexibility and malleability, higher porosity of BNC membrane	Sheet facial mask	[112]	2020
-	BNC carbide used as the positive electrode of a battery included in the facial mask; improved penetration of active ingredients into tissues	Sheet facial mask with a battery	[121]	2019
Cholinium-based ILs paired with anions derived from phenolic acids (caffeic, ellagic and gallic)	BNC membrane with increased re-hydration ability; slow and sustained release of active compounds; antioxidant and anti-inflammatory activities	Sheet facial mask	[114]	2019
Cholinium-based ILs paired with vitamins B anions	BNC membrane with reduced brittleness, increased re-hydration ability; fast release of active compounds	Sheet facial mask	[115]	2020
HA	Method to produce BNC–HA composite for facial masks preparation	Sheet facial mask	[117]	2017
Sericin–HA	Method to produce BNC–sericin–HA composite for facial masks preparation	Sheet facial mask	[118]	2018
HA–Rutin	Increased mechanical resistance of HA-MNs. Effective BNC controlled release of rutin. Maintenance of rutin antioxidant activity upon MNs system storage at room temperature for 6 weeks	MNs system for skin care	[119]	2021
PEGDA	BNC-3% and 5% PEGDA composites harder but less brittle than BNC gel; similar viscoelastic behavior to that of BNC gel	Hydrogel for facial masks	[122]	2017
PVA	BNC–PVA composite in a freeze-dried state; reduced possibility of contamination due to the freeze-dried nature of the BNC–PVA composite; lightweight and good swelling rate within 30 min	Freeze-dried additive for facial masks	[123]	2018
Active cosmetic formulations: anti-aging/lifting/purifying and regenerative	Non-invasive protocol for in vivo evaluation of the effectiveness and acceptance of BNC facial masks as delivery system of active substances	Sheet facial mask	[97]	2020
	**BNC AS A SUPPORT FOR THE IMMOBILIZATION OF ENZYMES**			
Papain	Oxidized BNC membrane with covalently immobilized papain; a higher amount of enzyme immobilized than in non-oxidized membrane; 93.1% recovered activity of the enzyme after immobilization	Enzyme-based skin care	[128]	2020
	**BNC AS EMULSION STABILIZER**			
-	TEMPO-oxidized BNC nanofibrils with reduced size; better stabilization of oil–water emulsions interface than with BNC (over 8 months)	Emulsion stabilizer	[132]	2016
-	Simple and easy scalable one-pot method to obtain TEMPO-oxidized BNC nanofibrils; the procedure assures the total removal of reactants; maintenance of the BNC nanofibrous structure and water-absorbing capacity.	Emulsion stabilizer	[12]	2019
Carboxymethyl cellulose (CMC)	Fully dispersible BNC:CMC dry formulation; better stabilizing effect of low oil-in-water emulsions than other dry commercial available celluloses; stabilization effect for up 90 days	Emulsion stabilizer	[134]	2020
	**BNC AS AN ALTERNATIVE TO MICROPLASTICS**			
-	BNC powder with 95% minimum purity, a size between 0.1 and 1 mm and a maximum residual water content of 15%; shape preservation and no-water absorption ability in liquid phase	Alternative to microplastics	[139]	2019
